# Impact of the Epigenetically Regulated Hoxa-5 Gene in Neural Differentiation from Human Adipose-Derived Stem Cells

**DOI:** 10.3390/biology10080802

**Published:** 2021-08-19

**Authors:** Rosa Hernández, Cristina Jiménez-Luna, Raúl Ortiz, Fernando Setién, Miguel López, Gloria Perazzoli, Manel Esteller, María Berdasco, Jose Prados, Consolación Melguizo

**Affiliations:** 1 Center of Biomedical Research (CIBM), Institute of Biopathology and Regenerative Medicine (IBIMER), University of Granada, 18100 Granada, Spain; r_faraya@hotmail.com (R.H.); crisjilu@ugr.es (C.J.-L.); roquesa@ugr.es (R.O.); gperazzoli@ugr.es (G.P.); melguizo@ugr.es (C.M.); 2 Biosanitary Institute of Granada (ibs.GRANADA), SAS-University of Granada, 18014 Granada, Spain; 3 Department of Anatomy and Embryology, Faculty of Medicine, University of Granada, 18071 Granada, Spain; 4 Cancer Epigenetics and Biology Program, Bellvitge Biomedical Research Institute, L’Hospitalet de Llobregat, 08908 Barcelona, Spain; fsetien@carrerasresearch.org (F.S.); mlopez@carrerasresearch.org (M.L.); mesteller@carrerasresearch.org (M.E.); mberdasco@carrerasresearch.org (M.B.); 5 Cancer Epigenetics Group, Cancer and Leukemia Epigenetics and Biology Program (PEBCL), Josep Carreras Leukemia Research Institute (IJC), 08916 Barcelona, Spain; 6 Epigenetic Therapies Group, Experimental and Clinical Hematology Program (PHEC), Josep Carreras Leukemia Research Institute, 08916 Barcelona, Spain

**Keywords:** mesenchymal stem cells, neuronal differentiation, epigenetic changes, *Hoxa-5*, CRISPR/dCas9

## Abstract

**Simple Summary:**

A deep knowledge of the regulation of genes involved in human adipose-derived mesenchymal stem cells (hASCs) neuronal differentiation is essential to their application in neurological disorder treatment. hASCs were induced to neuronal differentiation using three differentiation protocols and modulation of specific neuronal biomarkers and epigenetic genes changes were determined. An excellent neuronal differentiation of hASCs was obtained after Neu1 media exposure accompanied by relevant epigenetic changes in six genes including Hoxa-5. Moreover, functional analysis overexpressing the Hoxa-5 gene by CRISPR/dCas9 and lentiviral systems induced neuronal differentiation in hASCs which was improved and accelerated with the use of Neu1 media. These results suggest that Hoxa-5 plays a crucial role in the differentiation process, highlighting it as a potential candidate for the development of therapeutic strategies aimed at cell therapy in diseases related to the nervous system.

**Abstract:**

Human adipose-derived mesenchymal stem cells (hASCs) may be used in some nervous system pathologies, although obtaining an adequate degree of neuronal differentiation is an important barrier to their applicability. This requires a deep understanding of the expression and epigenetic changes of the most important genes involved in their differentiation. We used hASCs from human lipoaspirates to induce neuronal-like cells through three protocols (Neu1, 2, and 3), determined the degree of neuronal differentiation using specific biomarkers in culture cells and neurospheres, and analyzed epigenetic changes of genes involved in this differentiation. Furthermore, we selected the *Hoxa-5* gene to determine its potential to improve neuronal differentiation. Our results showed that an excellent hASC neuronal differentiation process using Neu1 which efficiently modulated NES, CHAT, SNAP25, or SCN9A neuronal marker expression. In addition, epigenetic studies showed relevant changes in *Hoxa-5*, *GRM4*, *FGFR1*, *RTEL1*, *METRN*, and *PAX9* genes. Functional studies of the *Hoxa-5* gene using CRISPR/dCas9 and lentiviral systems showed that its overexpression induced hASCs neuronal differentiation that was accelerated with the exposure to Neu1. These results suggest that *Hoxa-5* is an essential gene in hASCs neuronal differentiation and therefore, a potential candidate for the development of cell therapy strategies in neurological disorders.

## 1. Introduction

Human adipose-derived mesenchymal stem cells (hASCs) show clear advantages over other MSCs, such as low donor morbidity, minimal postoperative discomfort, and high cell yield. In fact, large amounts of hASCs can be obtained from liposuction surgery, since hASCs are five to eight times more numerous in fat than in other tissues [1]. The hASCs can be used not only for differentiation into mesodermal lineage but also into ectodermal and endodermal-derived tissues, including nervous tissue [2,3,4]. Despite variations in composition and duration of neuronal differentiation protocols exist, the assessment of differentiation effectiveness includes the analysis of specific neuronal markers such as Nestin (NES), Tubulin-III (TUB-III), microtubule-associated protein-2 (MAP2), enolase (ENS), and tyrosine hydroxylase (TH) [5], glial markers such as glial fibrillary acidic protein (GFAP) [6] and neuronal functional markers such as choline O-acetyltransferase (CHAT) and synaptosomal-associated protein-25 (SNAP25) [7]. In fact, using a differentiation protocol containing epidermal growth factor (EGF), fibroblast growth factor (FGF), and adenosine 3′,5′-cyclic monophosphate (cAMP), Urrutia et al. [8] observed a significant increase in NES and TUB-III in differentiated MSCs from a variety of sources. Dave et al. [9] found a significant increase in GFAP and TUB-III in hASCs treated with a differentiation medium containing cAMP and EGF and Kang et al. [10] demonstrated an increase in CHAT, TUB-III, and MAP2 in cells exposed to differentiation protocols containing NGF and FGF.

The therapeutic efficacy and biosecurity of differentiated hASCs has been widely discussed. Gao et al. [11] recently showed that, with a differentiation protocol rich in neurotrophic factors, hASCs could be converted into motoneuron-like cells that expressed a cohort of neuronal markers and that induced partial functional recovery when were transplanted into a spinal cord injury mouse model. Chudickova et al. [12] showed that the conditions of injured neural tissue with an inflammatory environment are an effective protocol for the differentiation of murine hASCs into cells expressing neuronal markers. In addition, clinical trials in neural system disorders have demonstrated positive results in terms of safety and effectiveness. In patients with multiple system atrophy, intrathecal hASCs transplantation was safe and well-tolerated [13]. Recently, the infusion of autologous hASCs has been demonstrated to be safe and feasible in patients with secondary progressive multiple sclerosis [14].

On the other hand, the coding sequences of hASCs and differentiated adult cells are almost the same [15], which seems to indicate that epigenetic factors play a primary role in determining hASCs fate and differentiation [16]. Pluripotency-related genes are hypermethylated in differentiated adult cells and hypomethylated in stem cells, indicating that DNA methylation participates in lineage determination [17]. In fact, epigenetic changes drive hASCs to commit to a particular lineage by repressing genes associated with the maintenance of stemness while expressing others related to differentiation into alternatives [18]. At present, epigenetic modifications that regulate hASCs functions and their adipogenic, osteogenic, chondrogenic, and neuronal differentiation remain unclear [19,20]. Regarding the induction of a neuronal phenotype from MSCs, it has been recently demonstrated that epigenetic reprogramming is required [21]. This plasticity could be the result of combining epigenetic modulating enzymes and specific signaling pathways [22]. The manipulation of hASCs towards a desired epigenetic status in order to transform them into the proper neuronal lineages could achieve a more durable and faster neuronal differentiation by itself or combined with enriched media. This epigenetic-based therapy is a promising tool under investigation for clinical application in several human conditions, including neurological diseases [23]. However, the mechanisms and essential genes that participate in the hASCs neuronal differentiation process are unknown. 

The hox gene family codifies transcription factors that contribute to determine the neuronal fate [24]. Concretely, *Hoxa-5* has been recently related to the differentiation of neurons from nuclei with autonomic functions in the mouse brain [25], and to the axon outgrowth of the posterior vagus motor neurons [26]. In addition, *Hoxa-5* is not only expressed in the early stages of development but also remains during adulthood, although the function in these stages is unknown [27]. Interestingly, the expression of *Hoxa-5* has been associated not only with the differentiation process of neurons but also with the dedifferentiation process in cancer (e.g., acute myeloid leukemia, colon cancer, or gastric cancer) [28].

The aim of this work was to determine and evaluate the importance of epigenetically-regulated genes in the neuronal differentiation process of hASCs. In this context, three differentiation protocols were tested to study epigenetic reprogramming. We further determined the most important genes involved in the process of neuronal differentiation and selected Hoxa-5 to verify its potential to improve and accelerate this process. These data might help to clarify the mechanisms of epigenetic interactions occurring during the neuronal differentiation of hASCs that are essential to maximizing the benefit and usefulness of these cells.

## 2. Materials and Methods

### 2.1. hASCs from Human Adipose Tissue, Neuronal Differentiation and Neurospheres Formation

This study was conducted in accordance with the Declaration of Helsinki. The study was approved by the ethical committee, under the project Fundació La Marató de TV3 (Ref:111430/31), code: 91432-N1-16 at the Andalusian Public Health System in Granada, and all participants provided informed consent allowing their anonymized information to be used for data analysis. Adipose tissue samples were obtained by minimally invasive liposuction procedures from three healthy patients aged 30 to 55 years. hASCs lines were extracted and characterized as described in our previous study [29]. hASCs were differentiated to neuronal lineage by in vitro induction using specific culture media. Three neuronal differentiation protocols, namely Neu1, Neu2, and Neu3, were employed. We chose these induction protocols based on the results their components showed in previous studies, with major changes observed in treated cells. Besides, different combinations of components were tested in our laboratory prior to our work. Neu1, Neu2, and Neu3 differentiation media (based on the procedure of Bossolasco et al. [30], Bae et al. [31], and Tondreau et al. [32] were used (see Supplementary Methods for detail: procedure of differentiation medium). Finally, hASCs were induced to forming neurospheres as previously described [33] (see Supplementary Methods for detail: procedure of neurospheres formation).

### 2.2. Immunofluorescence Analysis of Neuronal Markers

To determine the degree of neuronal differentiation, immunofluorescence assays were carried out. After the differentiation processes, the hASCs cells (plates of 12 and 24 wells) were fixed with 100% ethanol (20 min at −20 °C) and permeabilized with 0.1% Triton X-100 (Sigma Chemical Co) solution (10 min at RT). The cells were blocked with a blocking solution consisting of 5% goat serum (Sigma-Aldrich) and 0.3% Triton X-100 in 0.1% PBS-Tween (Bio-Rad) (1 h at RT). Following a PBS washing, cells were incubated with the primary antibodies (overnight at 4 °C): NES, GFAP, TUB-III, sodium voltage-gated channel alpha subunit-9 (SCN9A), synaptosome associated protein-25 (SNAP-25), O4 forkhead box (FOXO-4), CHAT, MAP2, TH, ENS, Tau protein (TAU), Neurofilament (NFM), and galactosylceramidase (GalC). Neurospheres were also incubated with n-cadherin and vimentin (Supplementary Table S1). Cells were incubated with secondary antibody (Alexa Fluor 488 goat anti-mouse IgG; A11001 Life Technology or bovine anti-mouse IgG-R; sc-2368 Santa Cruz) 1:500 dilution (1 h at RT). Nuclei were counterstained with Hoechst solution (Sigma). Negative controls that included the omission of primary or secondary antibodies were carried out and cells were examined under a fluorescence microscope (Nikon Eclipse E400). Images were generated using ImageJ software (version 1.52s). 

### 2.3. Quantitative RT-PCR

Analysis using mRNA was performed to determine the relative neuronal lineage gene expression levels. Total RNA was extracted using Trizol Reagent (Invitrogen, Carlsbad, CA, USA) and was converted into cDNA using a retro-transcriptase kit (Promega, Madison, WI, USA), following the manufacturer’s instructions. Specific quantitative RT-PCR primers were used (Supplementary Table S2).

### 2.4. Genome-Wide CpG Methylation Profiling

DNA samples from in vitro differentiated hASCs were extracted using conventional phenol:chloroform:isoamylalcohol (Sigma), quantified by Quant-iT PicoGreen dsDNA Reagent (Invitrogen), and DNA integrity was analyzed in a 1.3% agarose gel. Bisulfite modification of 600 ng genomic DNA was carried out with the EZ DNA Methylation Kit (Zymo Research, Irvine, CA, USA) following the manufacturer’s protocol. Next, 4 μL of bisulfite-converted DNA were used to hybridize on an Infinium HumanMethylation450 BeadChip, following the Illumina Infinium HD Methylation protocol. The chip was analyzed using an Illumina HiScan SQ fluorescent scanner and the intensities of the images were extracted using the GenomeStudio (2010.3) Methylation module (1.8.5) software. A three-step-based normalization procedure was performed using the Lumi package, available from Bioconductor [34] in the R statistical environment. This consisted of color bias adjustment, background level adjustment, and quantile normalization across arrays [24]. The methylation level (β) for each of the 485,577 CpG sites was calculated as the ratio of the methylated signal divided by the sum of methylated and unmethylated signals plus 100. To avoid batch effects, the function preprocess Funnorm in the minfi package was used [35]. It is particularly useful for studies comparing conditions with known large-scale differences, such as cancer/normal studies, or between-tissue studies. It has been shown that for such studies, functional normalization outperforms other existing approaches [36]. After the normalization step, probes related to X and Y chromosomes, and those containing SNPs with a frequency of >1% (1000 Genome Project) in the probe sequence or interrogated CpG sites were removed. Probes located in frequent copy number variant regions were also excluded. The methylation score of each CpG is represented as a β-value. DNA methylation microarray data are freely available for download from NCBI Gene Expression Omnibus under accession number GSE145614. DNA methylation values from undifferentiated hASCs were previously published [29], and free available from GEO under the accession number GSE33896 

### 2.5. Hierarchical Cluster Analysis and Definition of CpG Methylation Differences

Samples were clustered in an unsupervised manner using the 5000 most variable β-values for CpG methylation according to their standard deviation in the CpG sites located in promoter regions by hierarchical clustering. An agglomeration method for Manhattan distances was used. For the differential methylation analysis between conditions, Wilcoxon signed-rank tests were conducted in the R statistical environment for all CpGs. The resulting *p*-values were corrected for multiple testing [37]. The CpGs selected were those with adjusted values of *p* < 0.05 and an absolute methylation differential value of >0.33.

### 2.6. Bisulfite Genomic Sequencing of Multiple Clones

Once the candidate genes were selected, we determined their CpG island methylation status by PCR analysis of bisulfite-modified genomic DNA as described above. The PCR product was run on a 1% agarose gel and the bands were cut and purified using a purification kit (Macherey-Nagel, Düren, Germany) following the manufacturer’s instructions. Then, each genetic sequence of each sample was introduced in the pGEM-T Easy vector (Promega) cloned in *E. coli* bacteria and 12 clones of each were automatically sequenced to determine their degree of methylation. Primer sequences and annealing temperatures used are collected in Supplementary Table S2.

### 2.7. Generation of a Stable hASCs Line Overexpressing Hoxa-5 Gene by Lentivirus

To overexpress Hoxa-5 in hASCs, two exons (exon 1: 75-636 pb; exon 2: 1602-1847 pb) of the Hoxa-5 NM_019102 gene (Genome Browser) were isolated and amplified from a human lymphocytes cell line immortalized with the Epstein Barr virus, and the sequences of the restriction enzymes EcoRI and BamHI were added. After this, both exons were fused together and the PCR product was purified from a 1% agarose gel and digested with EcoRI and BamHI to be bound to the previously digested pLVX-IRES-Zsgreen vector (Takara Bio, Saint-Germain-en-Laye, France) in a 3:1 ratio. The product of the ligation was cloned in *E. coli* and sequenced. As a negative control, a Hoxa-5 truncated sequence was designed to eliminate the 180 nucleic bases of its homeobox. The sequence was ligated with the pLVX-IRES-tdTomato vector (Clontech) with red fluorescence, and finally, it was sequenced following the same methodology already described. Complete and truncated *Hoxa-5* gene sequences and primers used are shown in Supplementary Table S3. Lentivirus was packaged by transfecting HEK293T cells (cultured in the same conditions as the hASCs) using JetPrime reagent (Polyplus-Transfection SA, Illkirch-Graffenstaden, France) following the manufacturer’s instructions. Hoxa-5-pLVX-IRES-Zsgreen was cotransfected with the packaging vectors pMD2.G (envelope expressing plasmid, Addgene) and psPAX2 (packaging empty backbone, Addgene) in a 1:1:1 ratio. Transfection controls were carried out with the Truncated-Hoxa-5-pLVX-IRES-tdTomato, empty pLVX-IRES-Zsgreen, and empty pLVX-IRES-tdTomato vectors following the same methodology. Virus supernatant was collected 3 days after transfection and hASCs were infected using 0.45 µm-filtered fresh supernatant supplemented with 8 µg/mL polybrene. Twenty-four hours post-infection, the supernatant was replaced with standard DMEM medium supplemented with 10% FBS and 1% penicillin/streptomycin. Infected hASCs were passaged at a 1:1 ratio every 4 days using trypsin to favor the viral infection before using a cell sorter to select only transfected green or red fluorescent cells. To demonstrate the presence of the *Hoxa-5* gene in the infected cells quantitative RT-PCR assays were performed one week after the sorter. Total RNA was isolated from a 25 cm2 flask of infected cells using trypsin and mRNA levels were measured by RT-PCR using the SYBR-Green assay previously described (Supplementary Table S2).

### 2.8. Generation of a Hoxa-5—Expressing hASCs Cell Line Using a CRISPR/dCas9 System

pMLM3705 and MLM3636 plasmids were a gift from Keith Joung (Addgene plasmid #47754 and #43860, respectively). pdCAS9-NED was gently donated by Prof. Rots at UMCG [38]. Cloning of gRNAs was achieved as previously described [39]. Briefly, pairs of DNA oligonucleotides, called Hox1 and Hox2, encoding 20 nt gRNA targeting sequences were annealed together to create double-stranded DNA fragments with 4 bp overhangs. Primer sequences were: (Hox1, 5′-ACACCGTTCCGTGAGCGAGCAATTCG-3′ (sense) and 5′-AAAACGAATTGCTCGCTCACGGAACG-3′ (antisense); Hox2, 5′ACACCCGA AGTCGTACCCCATATTTG-3′ (sense) and 5′-AAAACAAATATGGGGTACGACTTCGG-3′ (antisense). These pairs were annealed together to generate short double-strand DNA fragments which were ligated into the MLM3636 plasmid (Addgene). The construction was cloned in *E. coli* competent bacteria and confirmed by DNA sequencing. To obtain large amounts of this plasmid for transfections, maxipreps (Qiagen) of bacteria grown in LB medium supplemented with ampicillin at a concentration of 50 µg/uL were made. To determine the activation of *Hoxa-5* in hASCs, combined transfections were carried out with the MLM3636-Hox1, MLM3636-Hox2, and the pMLM3705 plasmid (Addgene) with a dCas9-VP64 domain (VP64-*Hoxa-5*). A dCas9-NEF (non-effector) system (Groningen) was used as a negative control. Cotransfections of two CRISPR sequences (Hox1 and Hox2) and the plasmid with dCas9-VP64 or dCas9-NEF were carried out using lipofectamine (Invitrogen) at a 1:1:2 ratio, with 0.5 µg of total plasmid DNA concentration per well in a 24-well plate and 90% confluence. The transfection medium was removed and replaced with fresh complemented DMEM medium after 4 h and cells were collected two days after transfection. Total RNA was isolated using trypsin and mRNA levels were measured by quantitative RT-PCR using the SYBR-Green assay previously described.

### 2.9. Statistical Analysis

All of the analyses were carried out by triplicate, and the results were expressed as the mean ± standard deviation (SD). Statistical analysis was performed by the Student’s *t*-test (SPSS v.20, SPSS, Chicago, IL, USA). Values of *p* < 0.05 were considered significant.

## 3. Results 

### 3.1. Neuronal Differentiation of hASCs

**Figure 1:**
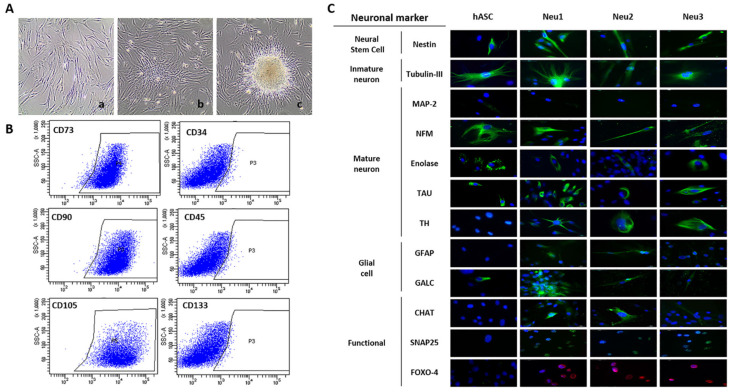
Isolation, characterization, and differentiation of hASCs. (**A**) The cells grow as a monolayer adhering to the surface of the flask and form colonies on (**a**) day 7, (**b**) day 14, and (**c**) day 21. (**B**) Immunophenotypic characterization of hASCs by FACScan analysis showing mesenchymal and hematopoietic surface markers. Approximately 99% of cells were positive for mesenchymal markers (CD73: 98.1%, CD105: 99.9%, and CD90: 99.6%), and 98.5% were negative for hematopoietic markers (CD45: 2.4%, CD34: 2.4%, and CD133: 1.7%), representing a typical mesenchymal-like immunophenotype (all mAbs from BD Biosciences). (**C**) Representative immunofluorescence analysis of markers in hASCs. Markers of neural stem cells, immature and mature neurons, glial cells, and functional markers (green) were analyzed in hASCs control, hASCs exposed to Neu1 (21 days), Neu2 (15 days), and Neu3 (10 days) media. Nuclei were stained with Hoechst solution (blue).

After cell isolation and monolayer establishment (Figure 1A), cells were characterized by flow cytometry and found to be positive for stem cell markers and negative for hematopoietic markers (Figure 1B) and exposed to the three neuronal differentiation protocols -Neu1, Neu2, and Neu3- (see Section 2). The immunofluorescence study (Figure 1C) revealed a significant increase in specific markers of neuronal stem cells (NES), young neurons (TUB-III), mature neurons (TAU, GFAP, and TH), and functional markers (SNAP25, CHAT, and FOXO4) in hASCs exposed to Neu1. To determine the proportion of differentiated cells after Neu1 exposure we analyze the number of cells that expressed TH and TAU and SNAP25, finding that 85 and 84% of the cells were positive for mature neuron markers (TH and TAU, respectively) and 79% were positive for the functional cell marker SNAP25. By contrast, little changes were observed with the use of Neu2 and Neu3, especially in functional markers, although Nestin and SNAP25 markers were significantly increased in Neu2-treated cells, and TH and SNAP25 markers in Neu3-treated cells.

**Figure 2:**
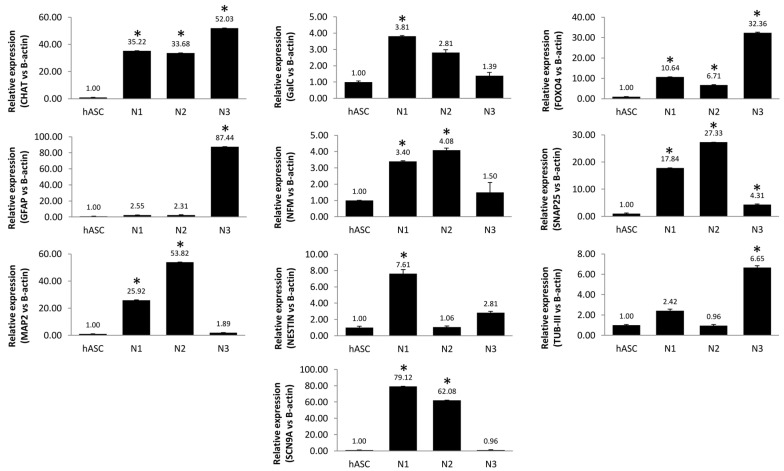
Quantitative RT-PCR analysis of markers in hASCs. Expression of neuronal markers was analyzed in hASCs control, hASCs induced with the Neu1 (21 days), Neu2 (15 days), and Neu3 (10 days) media. Statistically significant differences (Student’s *t*-test) (*) with respect to the control (*p* ≤ 0.05).

To assess these results, a quantitative RT-PCR (Figure 2) was carried out demonstrating that Neu1 was the differentiation protocol that induced a major change (7.6-fold) in NES and a significant change in MAP2 (25.92-fold) and SNAP25 (17.84-fold), CHAT (35.22-fold), FOXO4 (10.64-fold), and, especially, in SCN9A (79.12-fold), a marker related to the voltage-gated sodium channel. Hence, the modulation of the aforementioned markers suggests that hASCs are committed to the neuronal lineage. By contrast, the Neu2 did not induce a significant modulation of NES and produced a more subtle increase in NFM, MAP2, and SNAP25 than Neu1. There is an increase in the expression of CHAT (33.68-fold), FOXO4 (6.71-fold), SNAP25 (27.33-fold), MAP-2 (53.82-fold), and SCN9A (62.08-fold). Finally, Neu3 caused a large increase in the expression of astrocyte markers such as GFAP (87.44-fold), functional markers such as FOXO4 (32.36-fold), and CHAT (52.03-fold). In relation to the modulation of functional markers, the increase in SNAP2 was clearly corroborated by the three techniques in Neu1-treated cells. Furthermore, an increase in FOXO4 was observed by immunofluorescence and RT-PCR in all treated cells. A global evaluation of the neuronal markers modulation suggested that hASCs reached a neuronal lineage. Neu1 protocol was chosen as, overall, it induced the most significant changes in the protein expression of functional and mature neurons markers.

### 3.2. Neuronal Differentiation in hASCs Neurospheres

**Figure 3:**
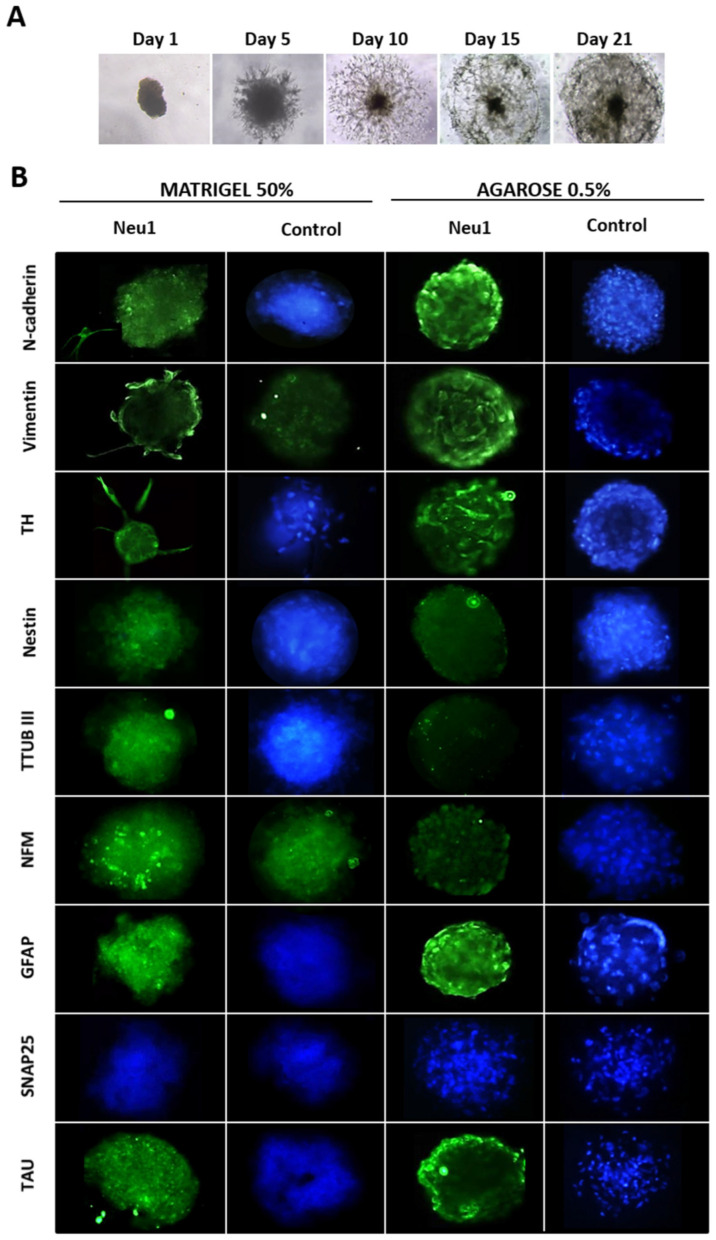
Differentiation of neurospheres from hASCs. (**A**) Representative optical microscope image of hASCs neurosphere formation (agarose 0.5%) after exposure to Neu 1 media (21 days). (**B**) Representative immunofluorescence images of marker expression (green) in hASCs neurospheres (matrigel 50% and agarose 0.5%) with and without Neu1 induction medium. The nuclei were stained with Hoechst solution (blue).

Based on the positive results with Neu1, a complementary study was carried out with neurospheres, tridimensional cultures that allow a higher interaction between hASCs (mimic in vivo differentiation process), and a more accurate evaluation of the differentiation ability of hASCs. Neu1-induced neurospheres grew, developing long extensions as dendrites until reaching a radius of approximately 250 to 350 μm (day 21 of culture), coincident with the maximum distance of oxygen diffusion (Figure 3A). In addition, an immunofluorescence study showed an increase in the expression of specific neuronal stem cell markers (similar to monolayer cultures) including TUB-III or mature neuron markers like TAU, NFM, and TH. However, a non-significant increase in functional markers such as SNAP25 was observed (Figure 3B). Interestingly, vimentin and N-cadherin expression, two cell adhesion molecules, increased in neurospheres suggesting a preliminary cell-cell contact, generally binding through calcium-dependent interactions that drive cells to self-assemble into aggregates, supporting their neural differentiation. 

### 3.3. DNA Methylation Changes Associated with Neuronal Differentiation of hASCs

**Figure 4:**
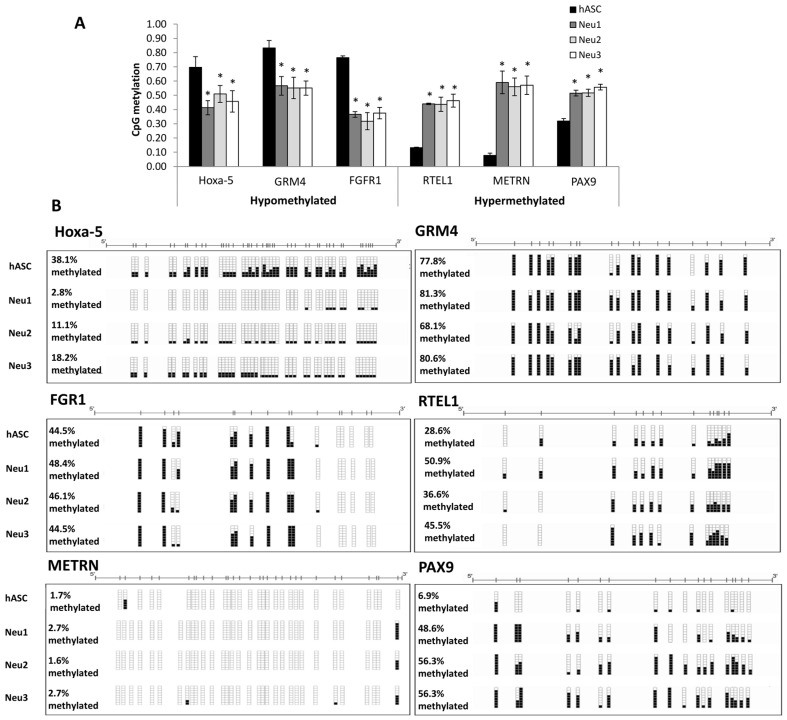
Promoter methylation studies of gene candidates. (**A**) Promoter methylation arrays of the Hoxa5, GRM4, FGFR1 (hypomethylated) and RTEL1, METRN, and PAX9 (hypermethylated). Statistically significant differences (Student’s *t*-test) (*) with respect to the control (*p* ≤ 0.05). (**B**) Validation of microarray results by bisulfite genomic sequencing of the promoter region of the same genes after differentiation with Neu1, Neu2, and Neu3. The results of bisulfite genomic sequencing of eight individual clones are shown, in which CpG dinucleotides are represented as short vertical lines. The presence of a methylated or unmethylated cytosine is indicated by a black or white square, respectively.

We analyzed the levels of DNA methylation of the human genome in hASCs obtained from different donors (*n* = 3) compared with neuronally-differentiated hASCs using the Neu1, Neu2, and Neu3 media. To search for specific changes in the methylation status of particular CpGs, we employed a threshold-based method using three replicates for each sample and a >20% CpG methylation β value as the cutoff. Six neurogenesis-related genes showing the greatest changes in hypomethylation (*Hoxa-5*, *GRM4*, *FGFR1*) and hypermethylation (*RTEL1*, *METRN*, *PAX9*) were detected. The genes selected were those with adjusted values of *p* < 0.05 and an absolute methylation differential value of >0.33 (Supplementary Table S4). As shown in Figure 4A, a decrease in methylation was observed in *Hoxa-5* when comparing control hASCs (≈70%) with differentiated cells (≈40%, 50%, and 45% in Neu1, Neu2, and Neu3, respectively). In addition, the methylation of *GRM4* and *FGFR1* represented ≈82% and ≈78% in control hASCs versus ≈55% and ≈37% respectively in differentiated cells, in the three differentiation protocols. On the other hand, a significant increase in the degree of methylation was found in *RTEL1*, *METRN*, and *PAX9* (≈45%, ≈55%, and ≈50% with Neu1; ≈45%, ≈52%, and ≈50% with Neu2; and ≈48%, ≈53%, and 52% with Neu3, respectively) in differentiated hASCs versus control cells (≈15%, ≈10%, and 35%, respectively) (Figure 4A). We further validated the microarray results using bisulfite genomic sequencing of multiple clones in differentially methylated candidate genes, obtaining the same results for the control and differentiated samples (Figure 4B), which were 77.8% for GRM4 and 44.5% for FGR1.

### 3.4. Modulation of Neuronal Markers in Stable hASC Line That Overexpresses the Hoxa-5 Gene

**Figure 5:**
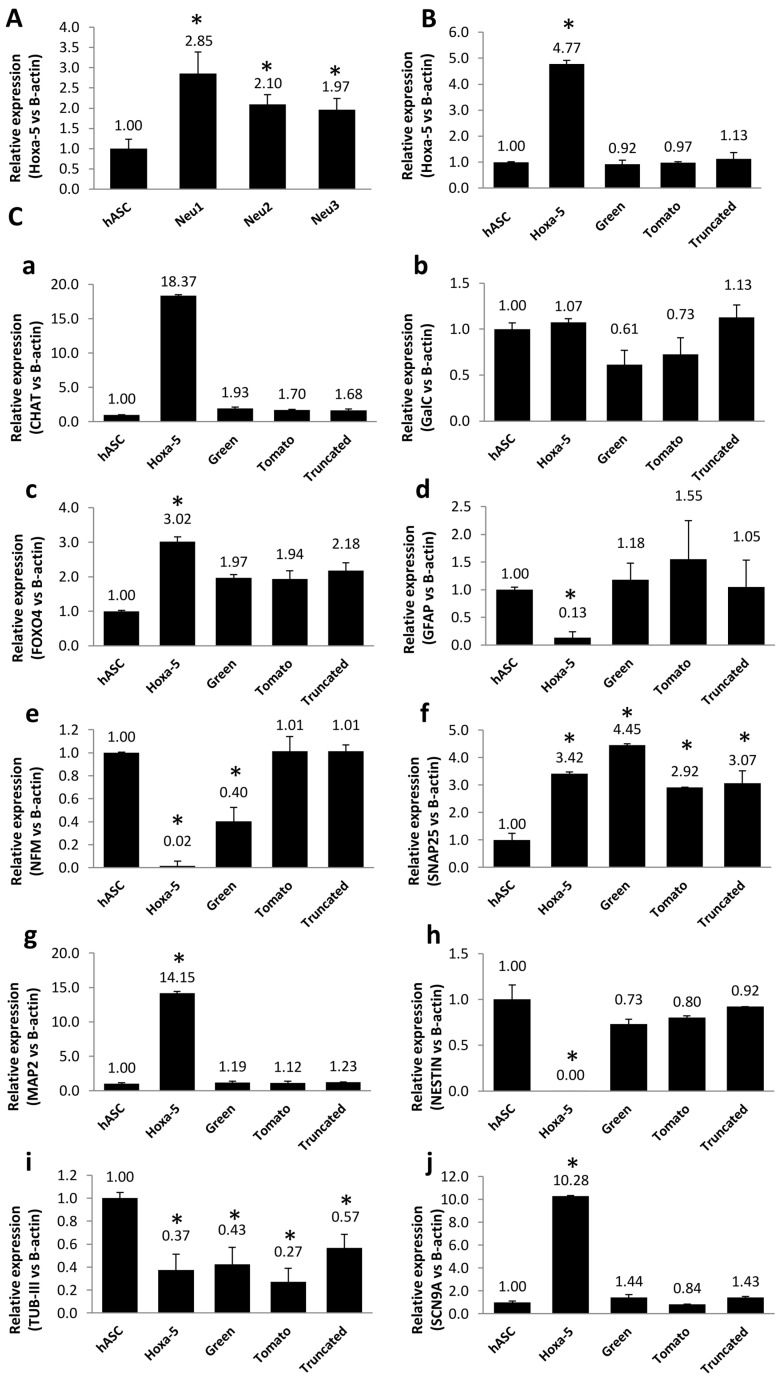
*Hoxa-5* expression after hASC exposure to differentiation media (Neu) and hASC transfection. (**A**) Quantitative RT- PCR of Hoxa-5 expression on differentiated hASC cells after Neu 1, 2, and 3 exposure. (**B**) Quantitative RT- PCR of Hoxa-5 expression after the use of a lentiviral system to obtain hASC-Hoxa-5-pLVX-IRES-Zsgreen (Hoxa-5 cells). hASC-Truncated-Hoxa-5-pLVX-IRES-tdTomato (Truncated cells), hASC-pLVX-IRES-Zsgreen (Green cells), and hASC-pLVX-IRES-tdTomato (Tomato cells) (see Section 2). (**C**) Neuronal markers expression (**a**: CHAT; **b**: GalC; **c**: FOXO4; **d**: GFAP; **e**: NFM; **f**: SNAP25; **g**: MAP2; **h**: Nestin; **i**: TUB-III; **j**: SCN9A) was analyzed in Hoxa-5, Truncated, Green, and Tomato cells by quantitative RT-PCR. Statistically significant differences (*) with respect to the control (*p* ≤ 0.05).

We selected the *Hoxa-5* gene to perform the functional study because of its high methylation and its crucial role in cell differentiation processes, as well as in the development of the central nervous system. Firstly, in order to know specifically the modulation of the Hoxa-5 expression after Neu1, 2, and 3 exposure, a study of transcripts was carried out by quantitative RT-PCR, revealing a significant increase in differentiated samples (Figure 5A). Secondly, to study the impact of the *Hoxa-5* gene in the process of neuronal differentiation, an hASC cell line that overexpressed this gene was generated through a lentiviral system (see Section 2). After infecting hASCs with HEK293T supernatant, the expression of *Hoxa-5* was determined by quantitative PCR (Figure 5B). To establish a functional consequence for the changes in the overexpression of the *Hoxa-5* gene after transfection, we studied the expression of neuronal markers in *Hoxa-5*, Green, Truncated, and Tomato cells by quantitative RT-PCR. As shown in Figure 5C, the specific neuronal stem cell marker MAP2 and the functional markers SCN9A and CHAT showed a significantly increased expression (14.15, 10.28, and 18.37-fold, respectively) in Hoxa-5 cells in comparison with control hASCs. In addition, some immature neuron markers such as NES and TUB III, and the glial marker GFAP, showed a significantly decreased expression. Controls with Truncated, Green, or Tomato cells did not show a significant modulation in the expression of neuronal markers. In conclusion, overexpression of the *Hoxa-5* gene involves an increased expression of other neuronal markers, ultimately enhancing neuronal differentiation. Although it is known that *Hoxa-5* participates in many tissue differentiation processes, the pathway by which these processes are carried out still needs to be clarified.

### 3.5. Neuronal Differentiation by Hoxa-5 Gene and Neu1 Medium in hASCs

**Figure 6:**
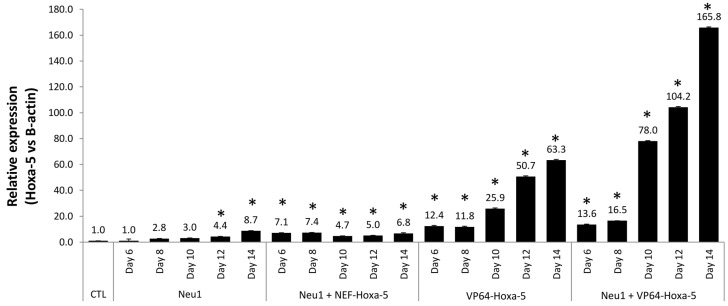
Modulation of Hoxa-5 expression in inducible hASCs generated by CRISPR/dCas9 system. The use of a CRISPR/dCas9 system allowed to obtain hASCs with overexpressed Hoxa-5 (VP64-Hoxa-5). NEF-Hoxa-5 cells (obtained with a non-functional CRISPR system) and hASCs were used as controls (see Section 2). All cells were exposed to Neu-1 (6, 8, 10, 12, and 14 days) to analyze the potentiation effect over Hoxa-5 expression. Analysis of the Hoxa-5 expression was carried out by RT-PCR. Statistically significant differences (*) with respect to the control (*p* ≤ 0.05).

To allow a stable transcriptional activation of the Hoxa-5 promoter and maximize its overexpression, an inducible cell line was generated using a CRISPR/dCas9 system, assaying the potentiation effect that the Neu1 neural differentiation protocol exerts. hASCs with overexpressed *Hoxa-5* by CRISPR/dCas9 were treated with the Neu1 differentiation medium (Neu1 + VP64-Hoxa-5) and compared to hASCs transfected with CRISPR/dCas9 without Neu1 induction (VP64-Hoxa-5). In addition, hASCs induced with Neu1, and hASCs induced with Neu1 and transfected with a non-functional CRISPR system (Neu1 + NEF-Hoxa-5), were used as controls. The study was taken up to 14 days to guarantee that the cell cultures kept an optimal condition and did not show any symptoms of aging or alterations due to transfection with lipofectamine. The study of mRNA by RT-PCR revealed increases of up to 8.7-fold in hASCs induced with Neu1 at day 14, and 63.3-fold in hASCs transfected with CRISPR/dCas9 without Neu1 induction, and also showed a synergistic effect (i.e., 165.8-fold increased expression) in hASCs treated with Neu1 + VP64-Hoxa-5 (Figure 6).

**Figure 7:**
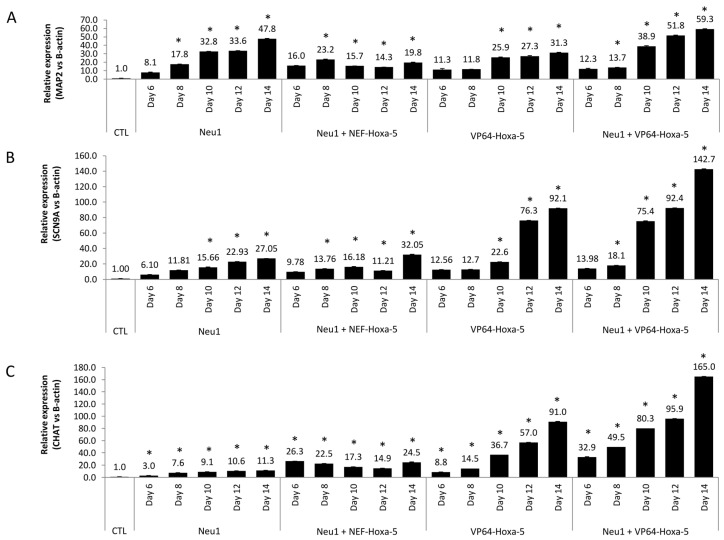
Modulation of neuronal markers in inducible hASCs generated by a CRISPR/dCas9 system. The use of a CRISPR/dCas9 system allowed to obtain hASCs with overexpressed Hoxa-5 gene (VP64-Hoxa-5). NEF-Hoxa-5 cells (obtained with a non-functional CRISPR system) and hASCs were used as controls (see Section 2). All cells were exposed to Neu-1 (6, 8, 10, 12, and 14 days) to analyze the potentiation effect of the neural differentiation process. Quantitative RT-PCR was used to evaluate the expression of MAP2 (**A**), SCN9A (**B**), and CHAT (**C**) markers in VP64-Hoxa-5 and VP64-Hoxa-5 exposed to Neu1 media. hASCs exposed to Neu1 media was used as a control. In addition, NEF-Hoxa-5 cells exposed to Neu1 media was also used as a control. Statistically significant differences (*) with respect to the control (*p* ≤ 0.05).

In order to determine the modulation in the expression of the neuronal markers MAP2, SCN9A, and CHAT, a quantitative RT-PCR was monitored (14 days) for all treatments. As shown in Figure 7, overexpression of *Hoxa-5* mediated by CRISPR/dCas9 enhanced the expression levels of mature neuronal markers such as MAP2 (which increased slightly), and functional markers of the nervous system such as SCN9A and CHAT. These increases were higher in CRISPR/dCas9-transfected cells (≈90-fold for SCN9A and CHAT, and ≈48-fold for MAP2 on day 14) than in Neu1-induced cells (≈27-fold for SCN9A, ≈11-fold for CHAT, and ≈47-fold for MAP2 on day 14). When both treatments were combined, a synergistic effect was observed in the expression of functional markers, reaching an increase of 142.7-fold for SCN9A, and 165-fold for CHAT on day 14 (*p* < 0.05). In summary, we confirmed that both the Neu1 differentiation medium and our designed Hoxa-5-CRISPR/dCas9 system enhance the neuronal differentiation of hASCs, showing a synergistic effect when applied together. 

## 4. Discussion

hASCs have shown promising results regarding the development of an alternative treatment for neurodegenerative diseases [40]. Several protocols can be used to induce hASCs into neuronal cells [41], although the control mechanisms underlying this differentiation process are far from being understood. Our aim was to determine an efficient protocol to drive hASCs towards neuronal-like cells and to perform a genome-wide epigenetic analysis of differentiated hASCs detecting modulations of relevant genes that may be used to improve this complex process. In this context, one of these genes (Hoxa-5) was selected to generate stable genetically modified hASCs and to induce a neural differentiation process.

We tested three different media named Neu1, Neu2, and Neu3 (see Section 2) to analyze the process of neuronal induction in hASCs. After 5 days, Neu1-containing EGF- induced hASCs to form neuron-like cells, with large spherical nuclei, a spindle-shaped body, and some extensions of dendrite-like cells. These cells showed a low proliferation rate. These findings were consistent with those from Garcez et al. [42], who demonstrated that EGF induces the differentiation of hASCs into a neuronal phenotype while FGF induces a glial phenotype. In fact, FGF seems essential to induce great functional differentiation towards the neural lineage, as Khademizadeh et al. recently showed in hASCs obtained from lipoaspirates [43]. In addition, Neu1 induced neurospheres formation, reaching a radius of approximately 250 to 350 μm, coinciding with the maximum oxygen diffusion distance [44]. By contrast, the Neu2 medium caused minor morphological changes in hASCs despite including hepatocyte growth factor, a neurotrophic factor that plays an important role in the survival of neurons in the brain [45], and vascular endothelial growth factor and EGF, which stimulate neuronal mesenchymal proliferation [31]. Finally, Neu3 containing cAMP, NGF, insulin, and 3-isobutyl-1-methylxanthine (IBMX), showed no morphological changes in hASCs despite Deng et al. [46] showing that cAMP may increase expression of neuronal markers such as GFAP, and Wang et al. [47] using IBMX to activate the protein kinase K, a crucial mediator in the neuronal differentiation of MSCs. On the other hand, the presence of NGF and insulin, two factors that improve proliferation, reduce apoptosis during differentiation, and/or are highly expressed in adult brains [48], was not effective in our hASCs differentiation. Immunofluorescence and quantitative RT-PCR analyses demonstrated an effective process of neuronal lineage induction of hASCs, especially after treatment with the Neu1 medium. In fact, the expression of TAU and TH, two mature neuronal markers, was increased in differentiated hASCs [49]. This last, TH, has been detected as an unequivocal sign of hASCs maturation towards dopaminergic cells [27]. In addition, the expression of NES and TUB-III, two markers of immature neurons also increased and some functional markers such as Snap25 and CHAT (synapse marker and cholinergic marker, respectively) [7], showed less expression changes. Finally, FOXO4, which is necessary for embryonic neuronal development and specifically critical for the differentiation of stem cells into neuronal cells [50] was also significantly modulated. Therefore, our results showed that exposure to an adequate medium promoted changes towards a neuronal morphology (i.e., axon- and dendrite-like structures) in hASCs, and also showed a pattern of expression markers, including functional markers, related to a neuronal lineage. In this context, Neu1 induced a more complete neuronal differentiation than Neu2 and Neu3.

Nevertheless, the major limitation for the application of differentiated hASCs in neuronal disorders is the impossibility of ensuring their long-term stability once transplanted. Although it is known that the process of neuronal differentiation is largely controlled by epigenetic changes, the precise mechanism is not yet fully understood. We used DNA from hASCs treated with three neuronal differentiation media and untreated hASCs, in order to carry out a genome-wide methylation array and to define specific genes with significant epigenetic modifications [40]. Comparative analysis between differentiated hASCs and controls showed significant hypomethylation of *Hoxa-5*, *GRM4*, *FGFR1*, and significant hypermethylation of *RTEL1*, *METRN*, and *PAX9*. These epigenetic changes were corroborated by bisulfite genomic sequencing of multiple clones and RT-PCR. The critical role of the *Hoxa-5* gene in successive steps of the central nervous system formation during embryonic and fetal development was determinant to selecting this gene for the incoming experiences.

Functional studies focused on this gene developing stable hASCs lines that overexpressed it. Previous studies indicated that Hox proteins contribute to neuronal fate and muscle connectivity through controlling the expression patterns of cell surface receptors [51] and that, specifically *Hoxa-5*, may have a potential role in the establishment and plasticity of pre-cerebellar circuits during postnatal and adult life [24]. In addition, Philippidou et al. [52] showed that *Hox5* genes orchestrate the neurons in the development of the phrenic motor column through the deployment of temporally distinct wiring programs, showing the importance of this gene in the correct development of motor neurons. Our results showed that *Hoxa-5* overexpression in hASCs positively modulated specific neuronal markers such as CHAT, FOXO4, MAP2, and SCN9A, in accordance with a process of neuronal differentiation. These results support the hypothesis that *Hoxa-5* is an essential gene in neuronal lineage and it is able to initiate a partial process of neuronal differentiation by itself [25]. Therefore, this gene could be a tool to improve the differentiation of hASCs to be applied in neurological disorders. In fact, when the CRISPR/dCas9-modified hASCs that overexpressed *Hoxa-5* were exposed to the Neu1 differentiation media, the expression of neuronal markers was significantly higher in comparison with a unique differentiation system

## 5. Conclusions

This study demonstrated that morphological and functional neuronal differentiation of hASCs obtained from lipoaspirates can be induced by using a specific combination of factors (Neu1), ensuring the appearance of a neuronal lineage and that this neuronal differentiation process implies at least a significant methylation change in six genes. Among them, *Hoxa-5* was able to induce neuronal differentiation, which was improved and accelerated by Neu1. Although future studies will be necessary to determine the relevance of other hypo- or hypermethylated genes in the neuronal lineage, our results suggest that *Hoxa-5* is an essential gene in this process. Therefore, *Hoxa-5* is a potential candidate for the development of therapeutic strategies aimed at cell therapy in diseases related to the nervous system.

## Data Availability

Publicly available datasets were analyzed in this study. This data can be found in NCBI Gene Expression Omnibus at https://www.ncbi.nlm.nih.gov/geo/ (accessed on 1 August 2021) under accession number GSE145614.

## Supplementary Materials

Supplementary information accompanies this paper at https://www.mdpi.com/article/10.3390/biology10080802/s1.
