# Supplementary material for: Impact of the Epigenetically Regulated Hoxa-5 Gene in Neural Differentiation from Human Adipose-Derived Stem Cells
Source: Biology (Basel). 2021 Aug 19;10(8):802. doi: 10.3390/biology10080802 (PMC8389620; doi:10.3390/biology10080802)
Supplement: Supplementary file 1 [file biology-10-00802-s001.zip › Supplementary methods.pdf]

Supplementary Methods

hASCs from human adipose tissue and neuronal differentiation

Neu1, Neu2 and Neu3 differentiation media (based in the procedure of Bossolasco et al. [23], Bae et al. [24] and Tondreau et al. [25] were used. For Neu1 induction [24], the hASCs were maintained in DMEM, supplemented with 25 µg/ml fibroblast growth factor (*FGF, Sigma-Aldrich*), 0.1 mg/ml epidermal growth factor (*EGF, Sigma-Aldrich*) and 10 µg/ml leukemia inhibitory factor (*LIF, Sigma*). The medium was changed every three days during 21 days. For Neu2 induction, hASCs were maintained in HAM-F12 medium (*Sigma-Aldrich*), supplemented with 20 ng/ml hepatocyte growth factor (*HGF, Sigma-Aldrich*), 20 ng/ml vascular endothelial growth factor (VEGF, Sigma-Aldrich) and 10 ng/ml epidermal growth factor. The medium was changed every three days during 15 days. For Neu3 induction based in the procedure of Tondreau et al., hASCs were maintained in neuronal progenitor basal medium (NPBM, Lonza), supplemented with 10 mg/ml adenosine 3′,5′-cyclic monophosphate (*AMPc, Sigma-Aldrich*), 25 ng/ml nerve growth factor (*NGF, Sigma-Aldrich*), 2.5 µg/ml insulin (*Sigma-Aldrich*) and 5 µM 3-isobutyl-1-methylxanthine (*IBMX, Sigma-Aldrich*) changing the medium every three days during 10 days. All cells were preserved at 37 °C in 95% humidity and 5% CO_2 atmosphere. All media were supplemented with 1% penicillin/streptomycin solution, 1% ciprofloxacin (*Fresenius Kabi*).

hASCs neurospheres formation

hASCs were induced to forming neurospheres as previously described [26]. Briefly, when hASCs were approximately 90% confluent, they were harvested with trypsin/EDTA and plated in 6 wells plate at a concentration of 2.5 x 10^5 cells/ml in DMEM/F12 medium complemented with 2% B27 serum-free supplement (*Gibco*) during 7 days. Neurospheres were collected using a 100 µm pipette and platted into the 96-well of matrigel or agarose previously prepared, at a density of 1 neurosphere per well in 150 µl Neu1 medium or DMEM/F12 medium in the case of controls. Neurospheres were maintained for 4 days changing the DMEM medium every 2 days, at 37 °C in 95% humidity and 5% CO2 atmosphere in all cases. For the preparation of the agarose plates, agarose (*Sigma-Aldrich)* was dissolved in distilled water at a concentration of 0.5% and boiled to the boil for ten minutes. 100 µl of liquid agarose was added to each well of 96-wells plate and kept at 37ºC for 30 minutes. After drying the agarose was covered with 150 µl of medium. For matrigel dishes, matrigel (*BD-Biosciences*) was dissolved in distilled water at a concentration of 50% and 100 µl of the liquid mixture was added to the 96-wells plate. After drying 30 minutes at 37ºC, the matrigel was covered with 150 µl of medium.
