# Supplementary material for: Impact of the Epigenetically Regulated Hoxa-5 Gene in Neural Differentiation from Human Adipose-Derived Stem Cells
Source: Biology (Basel). 2021 Aug 19;10(8):802. doi: 10.3390/biology10080802 (PMC8389620; doi:10.3390/biology10080802)
Supplement: Supplementary file 1 [file biology-10-00802-s001.zip › Supplementary Table S1.pdf]

Table S1. mAb antibodies used in immunofluorescence (IF) and western blot (WB).

mAbs	IF/WB Dilution	Reference
sodium voltage-gated channel alpha subunit-9 (SCN9A)	1:50/1:400	*Na+ CP type IXa Antibody H-17, sc-130096; Santa Cruz*
synaptosome associated protein-25 (SNAP-25)	1:200/1:400	*SNAP-25 Antibody C-18, sc7538; Santa Cruz*
tubulin-III (TUB-III)	1:200/1:1000	*Anti-tubulin Antibody beta III isoform, MAB1637; Millipore*
nestin (NES)	1:100/1:500	*Nestin Antibody, sc-23927; Santa Cruz*
choline O-acetyltransferase (CHAT)	1:200/1:400	*Choactase antibody E-7, sc-55557; Santa Cruz*
glial fibrillary acidic protein (GFAP)	1:50/1:500	*GFAP Antibody GA-5, sc-58766; Santa Cruz*
microtubule associated protein-2 (MAP2)	1:50/1:500	*MAP2 antibody A-4, sc74421; Santa Cruz*
O4 forkhead box (FOXO-4)	1:200/1:100	*Anti-FOXO-4, sab4501887; Sigma*
tyroxine hydroxylase (TH)	1:2500/1:4000	*Anti-tyrosine hydroxylase antibody, T2928; Sigma*
enolase (ENS)	1:100/1:400	*Enolase 5G10, sc-51882; Santa Cruz*
Tau protein (TAU)	1:50/1:100	*Anti-Tau antibody TAU-5, ab3931; ABCAM*
Neurofilament (NFM)	1:100/1:800	*Anti-Neurofilament antibody, 05744; Millipore*
galactosylceramidase (GalC)	1:50/1:500	*Anti-GalC antibody, mab342; Millipore*
n-cadherin	1:40/-	*N-cadherin antibody, sc-7939; Santa Cruz*
vimentin	1:50 /-	*Vimentin antibody, sc6260; Santa Cruz*
β-actin	-/1:10000	*β-actin antibody,, A3854, Sigma Aldrich*
