# Supplementary material for: Impact of the Epigenetically Regulated Hoxa-5 Gene in Neural Differentiation from Human Adipose-Derived Stem Cells
Source: Biology (Basel). 2021 Aug 19;10(8):802. doi: 10.3390/biology10080802 (PMC8389620; doi:10.3390/biology10080802)
Supplement: Supplementary file 1 [file biology-10-00802-s001.zip › Supplementary Table S2.pdf]

Table S2. Primer sequences

**Primers used in RT-PCR**				
**GENE**	**FORWARD**	**Tm**	**REVERSE**	**Tm**
**SCN9A**	5'-CTTCATCTATGGGGACATTC-3'	58.4	5'-GTCTTTTTGTCTGCATAGTAGG-3'	57.2
**SNP25**	5'-AAGGCTGATTCCAACAAAAC-3'	60.6	5'-GAAAAGCATGAAGGAGCTATC-3'	59.5
**TUBIII**	5'-GCGTCTACTACAACGAGG-3'	5.8	5'-ACTCTGACCAAAGATGAAATTG-3'	59.7
**NESTIN**	5'-CACCTCAAGATGTCCCTC-3'	58.0	5'TTGGGGTCCTGAAAGC-3'	59.7
**CHAT**	5'-TCAGTTCTTTGTCTTGGATG-3'	57.8	5'-TGGAAGCCATTTTGACTATC-3'	59.2
**GFAP**	5'-GCAGATTCGAGAAACCAG-3'	58.4	5'-CACATCACATCCTTGTGC-3'	58.5
**MAP2**	5'-GAAGATTTACTTACAGCCTCG-3'	57.2	5'-GGTAAGTTTTAGTTGTCTCTGG-3'	56.2
**FOXO4**	5'-GATATGTATATGGAGAACCTGG-3'	56.5	5'-AGAGTAGATATGAACACGCC-3'	55.8
**NFM**	5'-GCTGCGTACAGAAAACTC-3'	56.0	5'-CCTTGGGTTTCTGAATCTTAC-3'	59.1
**GALC**	5'-ATATGCTTTAGGACGTGTTG-3'	56.8	5'-GAGGTGAAATGACCCTTAATAG-3'	58.2
**B-ACTIN**	5'-AGCCTCGCCTTTGCCGATCC-3'	65.0	5'-ACATGCCGGAGCCGTTGTCG-3'	65.0
**HOXA5**	5'-TTAAAAAATAAATGAGTTTTTATTTTG-3'	56.4	5'-ACCAACCCCCTCTCTACTACTA-3'	59.4

**Primers used in bisulfite genomic sequencing of multiple clones**				
**GENE**	**FORWARD**	**Tm**	**REVERSE**	**Tm**
**FGFR1**	5'-TGGAGTATCTGGCCTCCAAG-3'	60.2	5'- ACCCTTCTCTTCCTACAACCTA-3'	59.5
**GRM4**	5'-CGTGCCCTCAAGTGGAACTA-3'	59.7	5'-GGCGGATGATCTTGTCGAAC-3'	59.1
**HOXA5**	5'-CAACCCCAGATCTACCCCTG-3'	59.2	5'-GAACTCCTTCTCCAGCTCCA-3'	59.7
**EN1**	5'-AGCAGCCTCTCGTATGGC-3'	59.2	5'-CTCCGTGATGTAGCGGTTTG-3'	59.0
**METRN**	5'-CTTTGAGCTGCGCGAGGA-3'	60.4	5'-AGACTCCTGCAGCTCCAC-3'	58.6
**PAX9**	5'-GAACGGGTTGGAGAAGGGAG-3'	60.0	5'-AGCAGCACTGTAGGTCATGT-3'	59.0
**RETL1**	5'-CGAGGAGGGAGGAAGAAGATC-3'	59.0	5'-TTCTTGGGGTCCTCAGCAAA-3'	59.2

**Primers used in the production of lentivirus and transfection of hASCs**				
**GENE**	**FORWARD**	**Tm**	**REVERSE**	**Tm**
**FGFR1**	5'-GGAAAGTTGAAATTTAAGGGAA-3'	59.6	5'-ACCCTTCTCTTCCTACAACCTA-3'	59.5
**GRM4**	5'-GAGGGTAAGTTTTGTGGAGAAT-3'	59.8	5'-ACCAACCAAAACACTAACCTTAA-3'	60.0
**HOXA5**	5'-TTAAAAAATAAATGAGTTTTTATTTTG-3'	56.4	5'-ACCAACCCCCTCTCTACTACTA-3'	59.4
**EN1**	5'-TGGTTTTGGGGTTATTTTTAG-3'	59.2	5'-AACCTAACTATTTTACACCCCC-3'	60.9
**METRN**	5'-GTTGTAGTTGGAGGGGTAGGTA-3'	60.6	5'-AACCRAATACAACCACTCAAC-3'	60.3
**PAX9**	5'-AGGGTTTTTGGAGTTTAATGGA-3'	62.0	5'-ACCAACCACTAACCACACAAAC-3'	62.5
**RETL1**	5'-TGTATGGGGGGTATTGAAATTT-3'	62.5	5'-AACTCCCATACCCCAAAAAAC-3'	62.4

Tm: melting temperatures in °C
