# Supplementary material for: Impact of the Epigenetically Regulated Hoxa-5 Gene in Neural Differentiation from Human Adipose-Derived Stem Cells
Source: Biology (Basel). 2021 Aug 19;10(8):802. doi: 10.3390/biology10080802 (PMC8389620; doi:10.3390/biology10080802)
Supplement: Supplementary file 1 [file biology-10-00802-s001.zip › Supplementary Table S3.pdf]

Table S4. Sequence of the Hoxa-5 wild type, sequence of Hoxa-5 truncated gene and protein and primer design and methodology employed for the construction of the transfection vectors.

Hoxa-5 gene sequence

ATGAGCTCTTATTTTGTAAACTCATTTTGCGGTCGCTATCCAAATGGCCCGGACTACCAGTTGCATAATTATGGAGATCA
TAGTTCCGTGAGCGAGCAATTCAGGGACTCGGCGAGCATGCACTCCGGCAGGTACGGCTACGGCTACAATGGCATGGA
TCTCAGCGTCGGCCGCTCGGGCTCCGGCCACTTTGGCTCCGGAGAGCGCGCCCGCAGCTACGCTGCCAGCGCCAGCGC
GGCGCCCGCCGAGCCCAGGTACAGCCAGCCGGCCACGTCCACGCACTCTCCTCAGCCCGATCCGCTGCCCTGCTCCGC
CGTGGCCCCCTCGCCCGGCAGCGACAGCCACCACGGCGGGAAAAACTCCCTAAGCAACTCCAGCGGCGCCTCGGCCG
ACGCCGGCAGCACCCACATCAGCAGCAGAGAGGGGGTTGGCACGGCGTCCGGAGCCGAGGAGGACGCCCCTGCCAG

PRIMER		SEQUENCE	Tm
EXON A	FORWARD	5'-AAAAAAGAATTCGCCGCCACCATGAGCTCTTATTTTGTAAACTCATTTTGCGGTC-3'	69.1
	REVERSE	5'-GCCTATGTTGTCATGACTTATGTGCAGCTTGCGCATCCA-3'	67.6
EXON B	FORWARD	5'-CACATAAGTCATGACAACATAGGCGGCCCGGAAGGC-3'	69.0
	REVERSE	5'-AAAAAAGGATCCTCAGGGACGGAAGGCCCCTCCTG-3'	69.1
EXON FUSION	FORWARD	5'-AAAAAAGAATTCGCCGCCACCATGAGCTCTTATTTTGTAAACTCATTTTGCGGTC-3'	69.1
	REVERSE	5'-AAAAAAGGATCCTCAGGGACGGAAGGCCCCTCCTG-3'	69.1

CAGCGAGCAGGCGAGTGCGCAGAGCGAGCCGAGCCCGGCGCCGCCCGCCCAACCCCAGATCTACCCCTGGATGCGCA
AGCTGCACATAAGTCATGACAACATAGGCGGCCCGGAAGGCAAAAGGGCCCGGACGGCCTACACGCGCTACCAGACC
CTGGAGCTGGAGAAGGAGTTCCACTTCAACCGTTACCTGACCCGCAGAAGGAGGATTGAAATAGCACATGCTCTTTGC
CTCTCCGAGAGACAAATTAAAATCTGGTTCCAAAACCGGAGAATGAAGTGGAAAAAAGATAATAAGCTGAAAAGCAT
GAGCATGGCCGCGGCAGGAGGGGCCTTCCGTCCCTGA

Primer complete hoxa-5 gene amplification

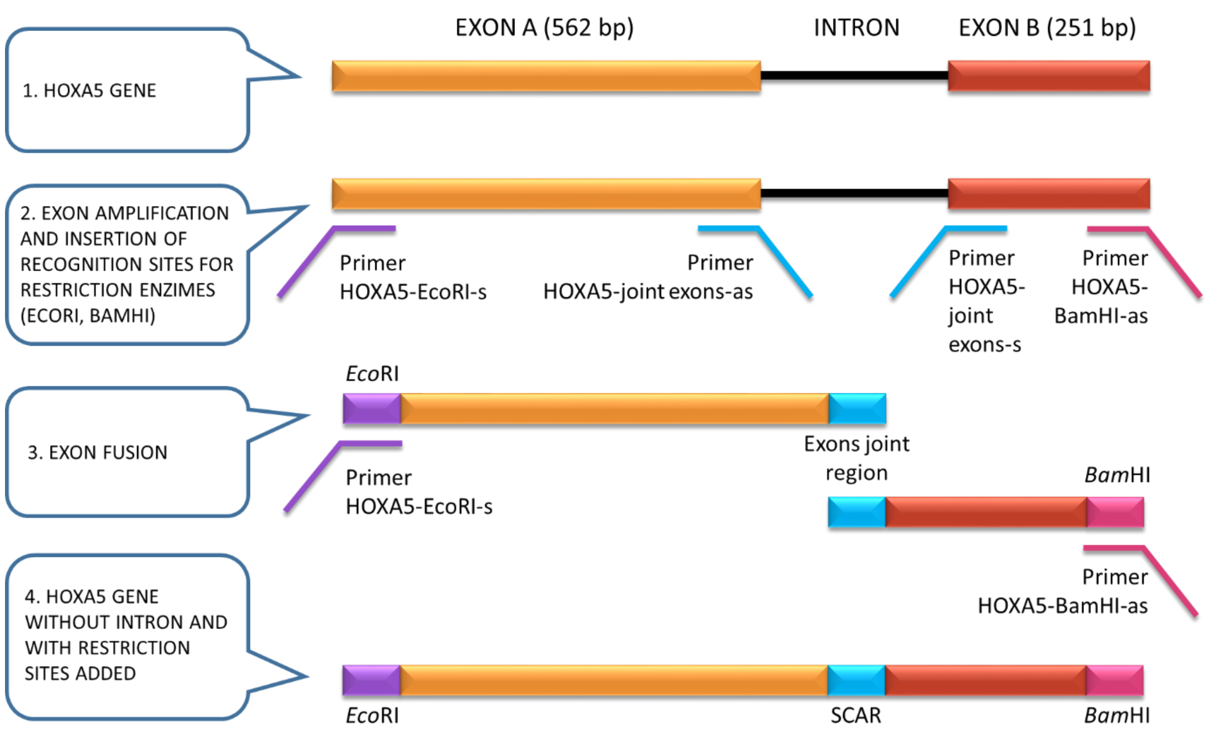

Hoxa-5 protein sequence
MSSYFVNSFCGRYPNGPDYQLHNYGDHSSVSEQFRDSASMHSGRYGYGYNGMDLSVGRSGSGHFGSGERARSYAASASAAPAE
PRYSQPATSTHSPQPDPLPCSAVAPSPGSDSHHGGKNSLSNSSGASADAGSTHISSREGVGTASGAEEDAPASSEQASAQSEPSPA
PPAQPQIYPWMRKLHISHDNIGG*PEGKRARTAYTRYQTLELEKEFHFNRYLTRRRRIEIAHALCLSERQIKIWFQNRRMKWKK*DNK
LKSMSMAAAGGAFRP (Homeobox underlined)

Truncated hoxa-5 gene sequence
ATGAGCTCTTATTTTGTAAACTCATTTTGCGGTCGCTATCCAAATGGCCCGGACTACCAGTTGCATAATTATGGAGATCAT
AGTTCCGTGAGCGAGCAATTCAGGGACTCGGCGAGCATGCACTCCGGCAGGTACGGCTACGGCTACAATGGCATGGATC
TCAGCGTCGGCCGCTCGGGCTCCGGCCACTTTGGCTCCGGAGAGCGCGCCCGCAGCTACGCTGCCAGCGCCAGCGCGGC
GCCCGCCGAGCCCAGGTACAGCCAGCCGGCCACGTCCACGCACTCTCCTCAGCCCGATCCGCTGCCCTGCTCCGCCGTG
GCCCCCTCGCCCGGCAGCGACAGCCACCACGGCGGGAAAAACTCCCTAAGCAACTCCAGCGGCGCCTCGGCCGACGCC
GGCAGCACCCACATCAGCAGCAGAGAGGGGGTTGGCACGGCGTCCGGAGCCGAGGAGGACGCCCCTGCCAGCAGCGA
GCAGGCGAGTGCGCAGAGCGAGCCGAGCCCGGCGCCGCCCGCCCAACCCCAGATCTACCCCTGGATGCGCAAGCTGCA
CATAAGTCATGACAACATAGGCGGCGATAATAAGCTGAAAAGCATGAGCATGGCCGCGGCAGGAGGGGCCTTCCGTCC
CTGA

Primer truncated hoxa-5 gene amplification

Primer		Sequence	Tm
FRAGMENT 1	FORWARD	5'-AAAAAAGAATTCGCCGCCACCATGAGCTCTTATTTTGTAAACTCATTTTGCGGTC-3'	69.1
	REVERSE	5'-GCTTATTATCGCCGCCTATGTTGTCATGACTTATGTGC-3'	65.5
FRAGMENT 2	FORWARD	5'-CATAGGCGGCGATAATAAGCTGAAAAGCATGAGCATGGCC-3'	68.6
	REVERSE	5'-AAAAAAGGATCCTCAGGGACGGAAGGCCCCTCCTG-3'	69.1
FRAGMENT FUSION	FORWARD	5'-AAAAAAGAATTCGCCGCCACCATGAGCTCTTATTTTGTAAACTCATTTTGCGGTC-3'	69.1
	REVERSE	5'-AAAAAAGGATCCTCAGGGACGGAAGGCCCCTCCTG-3'	69.1

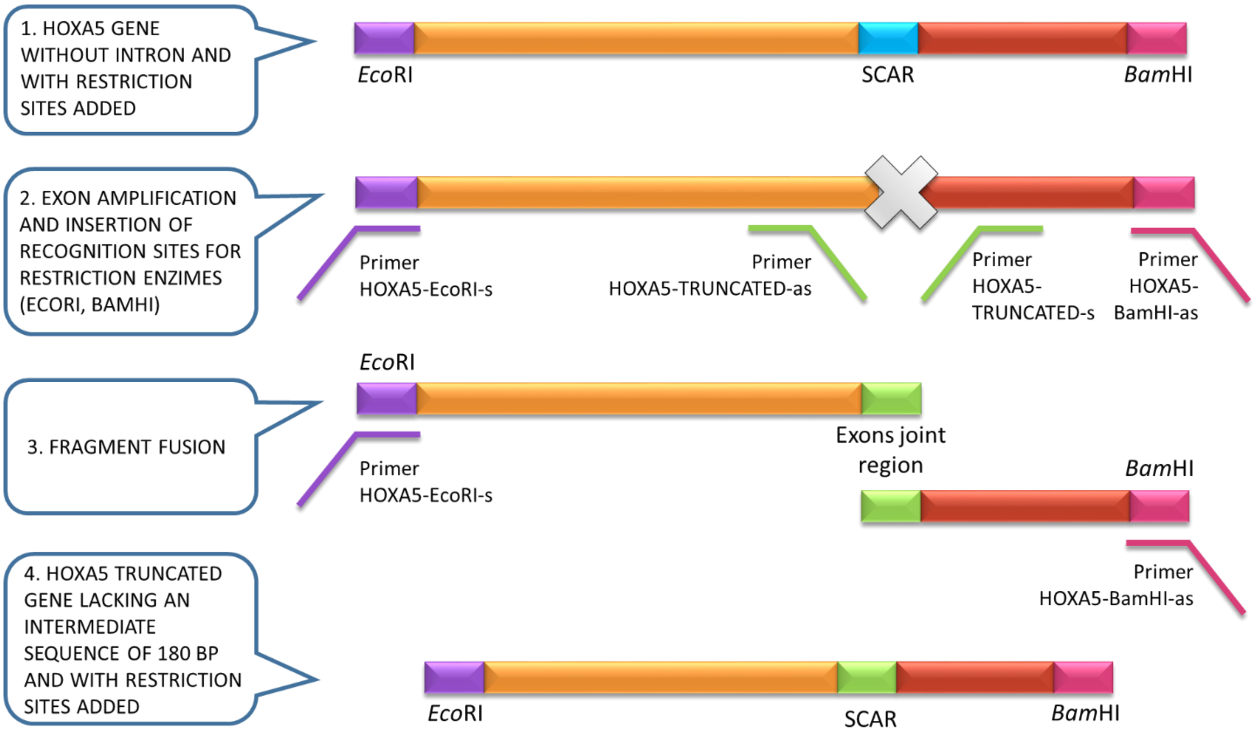

Hoxa-5 truncated protein sequence

MSSYFVNSFCGRYPNGPDYQLHNYGDHSSVSEQFRDSASMHSGRYGYGYNGMDLSVGRSGSGHFGSGERARSYAASASAAPAE
PRYSQPATSTHSPQPDPLPCSAVAPSPGSDSHHGGKNSLSNSSGASADAGSTHISSREGVGTASGAEEDAPASSEQASAQSEPSPA
PPAQPQIYPWMRKLHISHDNIGGDNKLKSMSMAAAGGAFRP
